# Effects of Shape and Position of Gingival Margin of Maxillary Anterior Teeth on Perception of Smile Aesthetics by Periodontists, Restorative Dentists, General Dentists, and Non-professionals 

**DOI:** 10.30476/dentjods.2025.102193.2344

**Published:** 2025-09-01

**Authors:** Moien Masoumi, Fahimeh Rashidi Maybodi, Maryam Sabet, Farnaz Farahat

**Affiliations:** 1 Student Dentistry, School of Dentistry, Shahid Sadoughi University of Medical Sciences, Yazd, Iran.; 2 Dept. of Periodontics, School of Dentistry, Shahid Sadoughi University of Medical Sciences, Yazd, Iran.; 3 Dept. of Periodontics, School of Dentistry, Shiraz University of Medical Sciences, Shiraz, Iran.; 4 Dept. of Restorative Dentistry, School of Dentistry, Shahid Sadoughi University of Medical Sciences, Yazd, Iran.

**Keywords:** Aesthetics, Gingiva, Perception, Periodontist, Restorative Dentist, Smiling

## Abstract

**Background:**

Each patient may have a different idea of a beautiful smile. Also, during treatment, periodontists, restorative dentists and general dentists should be aware that their personal views on the beauty of a smile and their degree of sensitivity in the perception of beauty changes may differ, and this difference may affect the treatment process.

**Purpose:**

This research seeks to evaluate how alterations in the shape and position of the gingival margin in maxillary anterior teeth influence the perception of smile aesthetics among different groups including periodontists, restorative dentists, general dentists, and non-professionals.

**Materials and Method:**

This cross-sectional study was conducted on 60 raters in four groups (n= 15) of periodontists, restorative dentists, general dentists, and non-professionals. The raters received 10 photographs including one original and 9 altered images, and they were asked to rate them regarding smile aesthetics. Data were analyzed by the Kruskal-Wallis, Fisher’s exact, and t-test (alpha=0.05).

**Results:**

All four rater groups gave the highest score to the original image and the lowest score to the asymmetrical changes on shape and position of the gingival zenith; the statistical difference in this regard was significant in the group of periodontists, restorative dentists, and general dentists (*p*< 0.05) but not in non-professionals (*p* Value> 0.05). Males were more sensitive than females in the detection of changes in shape and position of the gingival margin; however, the statistical difference was only significant for images showing an asymmetrical change on the right or left side (*p*< 0.05).

**Conclusion:**

A significant difference in opinion was seen among periodontists, restorative dentists, and general dentists regarding the effect of the shape and position of the gingival margin of maxillary anterior teeth on the perception of smile aesthetics, highlighting the need to reach an interdisciplinary consensus prior to gingivectomy and aesthetic crown lengthening procedures.

## Introduction

Dental aesthetics is an important factor in facial attractiveness and plays a fundamental role in social interactions [ 1 ]. Smile aesthetics depends on the color, size, shape, and position of the teeth, upper lip position, tooth show, and gingival display [ 2 ]. To be more exact, smile arc, tooth size ratio, midline alignment, inclination of teeth, size of buccal corridor, gingival height and contour, and presence or absence of diastema determine the smile’s harmony, symmetry, and attractiveness [ 3 - 4 ]. All these parameters should be in harmony to create a beautiful smile [ 5 ]. 

Smile design is defined as the aesthetic reconstruction of teeth that show in a smile [ 6 ]. In the anterior of maxilla, the position of the gingival margin is an important parameter for achieving an ideal smile. An appropriate relationship between the periodontium and restoration is imperative for optimal health and aesthetics. Crown lengthening surgery may be required to prevent invasion of the restoration margin to the biologic width, to change the labial gingival profile or to attain the ideal tooth size when there is inadequate passive eruption or anatomical issues [ 7 ]. 

The gingival tissue surrounding the maxillary anterior teeth plays a fundamental role in the creation of a beautiful smile. The gingival zenith is defined as the most apical point of the scalloped gingival margin [ 8 ]. Gingival zenith and its spatial orientation mesiodistally and apicocoronally can serve as a valuable reference for the determination of smile aesthetics. Ideally, the gingival zenith of the maxillary central incisor and the gingival zenith of canine teeth are at the same level apicocoronally, while the gingival zenith of lateral incisors is 1 mm coronal to that of adjacent teeth. On mesiodistal aspect, the gingival zenith of central incisors has a slight shift towards the distal while the gingival zenith of the lateral incisor and the gingival zenith of the canine teeth are along the vertical midline of the teeth [ 9 ]. 

Aesthetic perception varies from one individual to the next. According to Nomura *et al*. [ 10 ], trained eyes of examiners more easily detect asymmetry. Since the opinions of specialists regarding facial and dental aesthetics may not be the same as the aesthetic expectations of patients, the role of different factors that may affect the perception of smile aesthetics must be investigated and explained to the patients before any treatment [ 11 ]. 

In a previous study, a midline deviation of 1 mm was considered unaesthetic by orthodontists, while this value was 2 mm for oral and maxillofacial surgeons. Also, only orthodontists paid attention to the golden ratio in anterior teeth [ 12 ]. A more recent study showed that dental specialists had lower aesthetic perception and were less sensitive to the presence or absence of anterior diastema than general dentists, dental students, and non-professionals [ 13 ]. However, another study reported a higher sensitivity of dentists than non-professionals to the presence of midline diastema [ 14 ]. 

The demand for cosmetic dental procedures and smile design has significantly risen in recent years. These treatments typically require a collaborative, multidisciplinary approach, often involving periodontists and restorative dentists. It is also essential to consider patient expectations and preferences during treatment. Given the ongoing debate about the differences in aesthetic perception between dental professionals and the general public, as well as the wide range of factors assessed in previous studies, this issue remains complex, so this study aimed to assess the effects of the shape and position of the gingival margin of maxillary anterior teeth on the perception of smile aesthetics by periodontists, restorative dentists, general dentists, and non-professionals to see if there is any difference among them or not. 

## Materials and Method

This cross-sectional study was conducted on 60 raters in four groups (n=15) of periodontists, restorative dentists, general dentists, and non-professionals. The non-professionals were selected among those referred to the Periodontology Department of Yazd Shahid Sadoughi University of Medical Sciences. The specialists were recruited from dentistry faculty members of both Shahid Sadoughi and Shiraz University of Medical Sciences. The study protocol was approved by the Ethics Committee of the University (IR.SSU.DENTISTRY.REC. 1402.014). 

### Sample size

The sample size was calculated to be 60 according to a previous study assuming 95% confidence interval, a standard deviation of the aesthetic perception score of 1.5, and an estimation error of 0.5 [ 13 ]. The raters were selected by convenience sampling method. 

### Data collection

**Figure 1:**
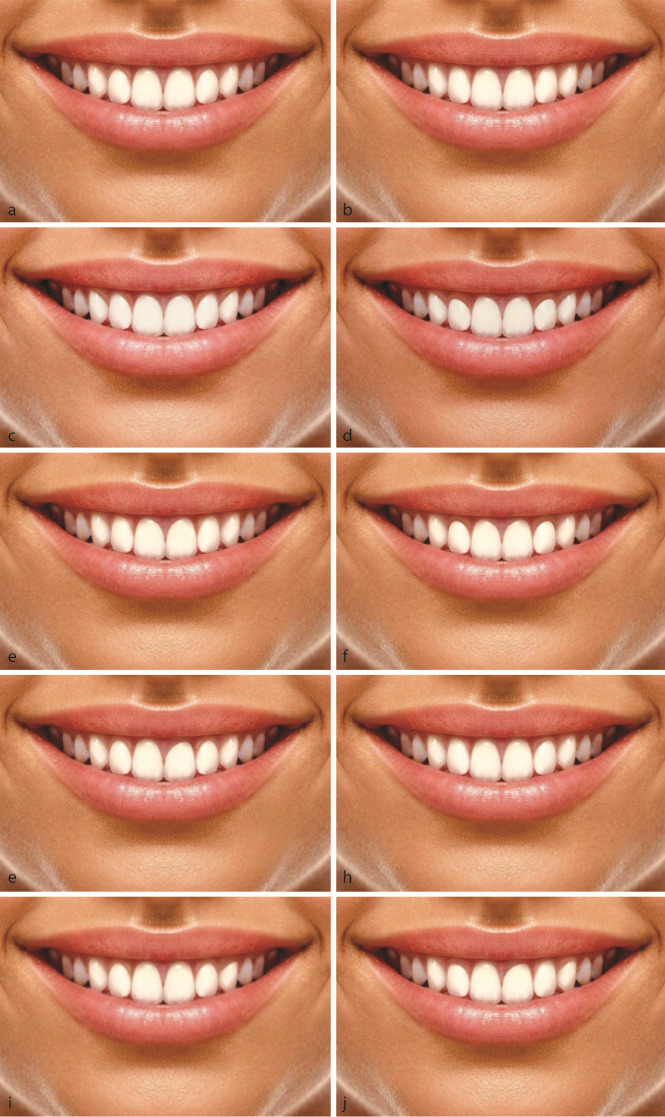
**a:** Normal size and position of gingival zeniths, **b:** The gingival zenith position of the lateral incisor was at the level of the central incisor and canine teeth bilaterally, **c:** The shapes of gingival zeniths of all teeth were 1 mm distal than normal and the position of the gingival zenith of the lateral incisor was at the level of central incisor and canine teeth bilaterally, **d:** The shapes of gingival zeniths of all teeth were 1 mm distal relative to the midline, and the positions of the gingival zeniths of all teeth were normal bilaterally, **e:** The shape of gingival zenith of the central incisor was 1 mm distal relative to normal while those of the lateral incisor and canine teeth were at the midline bilaterally, and the position of the gingival zenith of the lateral incisor was at the level of the central incisor and canine teeth, **f:** The shape of gingival zenith of the central incisor was 1 mm distal relative to the midline, while those of the lateral incisor and canine teeth were at the midline, and the positions of gingival zeniths of all teeth were normal bilaterally, **g:** The shape and position of the gingival zenith of the central incisor on one side were 1mm different from those of the contralateral central incisor, **h:** The shape and position of the gingival zenith of the lateral incisor on one side were 1mm different from those of the contralateral lateral incisor, **i:** The shape and position of gingival zenith of canine tooth of one side were 1 mm different from those of the contralateral canine tooth, **j:** The shape and position of the gingival zenith of all teeth on one side had a 1 mm difference with the shape and position of the gingival zenith of the teeth on the other side

A frontal-view extra-oral full-face photograph was obtained from a young woman with ideally aligned teeth and a beautiful smile and was digitally altered as follows, using Adobe Photoshop CS8 version 24 software [ 15 - 16 ]:

**Photograph A:** The shape of central incisor gingival zenith was 1 millimeter (mm) distal relative to the midline, the gingival zeniths of the lateral incisor and canine teeth were at the midline (at both sides), and the position of the gingival zenith of the lateral incisor was 1 mm coronal relative to the central incisor and canine teeth bilaterally (original photograph) (Figure 1a). 

**Photograph B:** The shapes of gingival zeniths of the central and lateral incisors and canine teeth were normal, and the gingival zenith position of the lateral incisor was at the level of the central incisor and canine teeth bilaterally (Figure 1b).

**Photograph C:** The shapes of gingival zeniths of all teeth were 1 mm distal relative to normal, and the position of the gingival zenith of the lateral incisor was at the level of central incisor and canine teeth bilaterally (Figure 1c).

**Photograph D:** The shapes of gingival zeniths of all teeth were 1 mm distal relative to the midline, and the positions of the gingival zeniths of all teeth were normal bilaterally (Figure 1d).

**Photograph E:** The shape of gingival zenith of the central incisor was 1 mm distal relative to normal, while those of the lateral incisor and canine teeth were at the midline bilaterally, and the position of the gingival zenith of the lateral incisor was at the level of the central incisor and canine teeth (bilaterally) (Figure 1e).

**Photograph F:** The shape of gingival zenith of the central incisor was 1 mm distal relative to the midline, while those of the lateral incisor and canine teeth were at the midline, and the positions of gingival zeniths of all teeth were normal bilaterally (Figure 1f).

**Photograph G:** The shape and position of the gingival zenith of the central incisor on one side were 1 mm different from those of the contralateral central incisor (Figure 1g). 

**Photograph H:** The shape and position of the gingival zenith of the lateral incisor on one side were 1 mm different from those of the contralateral lateral incisor (Figure 1h). 

**Photograph I:** The shape and position of gingival zenith of canine tooth of one side were 1 mm different from those of the contralateral canine tooth (Figure 1i).

**Photograph J:** The shape and position of the gingival zenith of all teeth on one side had a 1 mm difference with the shape and position of the gingival zenith of the teeth on the other side (Figure 1j). 

In the present study, the shape of gingival zenith was defined as the location of gingival zenith relative to the longitudinal axis, while the position of gingival zenith was defined as its apico-coronal position. Each image was coded, and the photographs were randomly arranged in a photo album. The four rater groups were provided with the photographs in the photo album and were asked to fill out the attached form, which asked for demographic information about the participants (age, gender, field, and level of education). The raters were also asked to rate the level of smile attractiveness of each of the 10 images by using a 10-score visual analog scale, with a score of 1 indicating the least attractive smile and a score of 10 indicating the most attractive smile. The raters were allowed to review the photographs again within a 2-minute period but were not allowed to compare the images by putting them next to each other. To evaluate the reliability of the assessments, 10 raters were randomly selected and were asked to rate the images again after a 2-week interval. 

Data were analyzed by SPSS 25 (SPSS Inc., IL, USA). The normal distribution of data was evaluated by the Shapiro-Wilk and Kolmogorov-Smirnov tests. Due to the non-normal data distribution (*p*< 0.05), comparisons were made by the Kruskal-Wallis test, t-test, and Fisher’s exact test at the 0.05 level of significance. 

## Results

Table 1 presents the kappa coefficient of agreement of the raters for different photographs. A total of 43 females (71.7%) and 17 males (28.3%) participated in this study. As shown in Table 2, a significant difference was present among the four rater groups in gender distribution (*p*= 0.02).

**Table 1:** Kappa coefficient of agreement of the raters for different photographs

Photograph	A	B	C	D	E	F	G	H	I	J
Kappa coefficient	-0.075	-0.076	-0.091	-0.037	-0.036	-0.062	-0.052	-0.083	-0.049	-0.015

**Table 2:** Gender distribution in the four rater groups

Gender Rater group	Female	Male	*p* Value*
Periodontists	53.3%	46.7%	0.02*
Restorative dentists	93.3%	6.7%	
General dentists	53.3%	46.7%	
Non-professionals	86.7%	13.3%	

*Fisher’s exact test

In the group of non-professionals, females allocated higher scores to all photographs than males, and this difference was significant for photographs A, E and J. The greatest difference was observed for the photograph A (original photograph) (*p*= 0.012, Table 3).

**Table 3:** Scores given to different photographs by male and female non-professionals raters

Photograph	Gender	Mean score	*p* Value*
A	Male	7.88	0.012
	Female	9.05	
B	Male	7.12	0.170
	Female	8.19	
C	Male	7.47	0.802
	Female	7.93	
D	Male	6.59	0.309
	Female	7.21	
E	Male	7.18	0.034
	Female	8.16	
F	Male	6.82	0.784
	Female	7.02	
G	Male	4.82	0.118
	Female	5.98	
H	Male	7.06	0.763
	Female	7.33	
I	Male	6.29	0.082
	Female	7.09	
J	Male	4.00	0.014
	Female	5.51	

*t-test

The mean age of the raters was 31.30±5.42 years. The mean age was 33.67±4.76 years for periodontists, 32.47±3.79 years for restorative dentists, 27.87±3.27 years for general dentists, and 31.20±7.50 years for non-professionals. The difference in the mean age was significant among the four groups (*p*= 0.019). In the group of non-professionals, the scores given to the photographs were analyzed according to age (Table 4), which revealed a significant difference only for the photograph J (*p*= 0.011) such that non-professional raters under 30 years of age gave a significantly higher score to this photograph than other age groups. 

**Table 4:** Scores given by the non-professionals raters of different age groups to different photographs

Photograph	Age (yrs.)	Mean score	*p* Value*
A	<30	8.50	0.285
	>31	8.93	
B	<30	7.67	0.624
	>31	8.10	
C	<30	7.57	0.478
	>31	8.03	
D	<30	7.00	0.958
	>31	7.07	
E	<30	7.77	0.640
	>31	8.00	
F	<30	7.33	0.084
	>31	6.60	
G	<30	6.10	0.112
	>31	5.20	
H	<30	7.33	0.446
	>31	7.17	
I	<30	7.23	0.072
	>31	6.50	
J	<30	5.80	0.011
	>31	4.37	

*t-test

Table 5 presents the mean scores given by four rater groups to different photographs. As shown by the Kruskal-Wallis test, a significant difference existed among the opinions of the rater groups regarding photograph A (*p*= 0.031), such that restorative dentists gave the highest score, and general dentists gave the lowest score to this photograph. A significant difference was also found among the opinions of the rater groups regarding photo graph E (*p*= 0.005), such that periodontists gave the highest score, and non-professionals gave the lowest score to this photograph. A significant difference was noted among the opinions of the rater groups regarding photograph G (*p*= 0.002), such that non-professionals gave the highest score, and restorative dentists gave the lowest score to this photograph. A significant difference existed among the opinions of the rater groups regarding photograph I (*p*= 0.005), such that non-professionals gave the highest score, and restorative dentists gave the lowest score to this photograph. A significant difference also existed among the opinions of the rater groups regarding photograph J (*p*= 0.018), such that non-professionals gave the highest score, and restorative dentists gave the lowest score to this photograph.

**Table 5:** Mean scores given by the four rater groups to different photographs

Photograph	Rater group	Mean	Standard deviation	Median	*p* Value*
A	Periodontists	9.07	0.961	9.00	0.031*
	Restorative dentists	9.33	0.816	9.00	
	General dentists	7.73	1.710	8.00	
	Non-professionals	8.73	1.223	9.00	
B	Periodontists	8.20	1.612	8.00	0.176*
	Restorative dentists	8.67	1.047	9.00	
	General dentists	7.13	2.295	9.00	
	Non-professionals	7.53	2.031	8.00	
C	Periodontists	8.20	1.373	8.00	0.332*
	Restorative dentists	8.33	1.397	9.00	
	General dentists	7.07	2.219	7.00	
	Non-professionals	7.60	2.063	8.00	
D	Periodontists	7.60	1.765	8.00	2.332*
	Restorative dentists	7.20	1.699	7.00	
	General dentists	6.27	1.944	7.00	
	Non-professionals	7.07	2.154	7.00	
E	Periodontists	8.67	1.175	9.00	0.005*
	Restorative dentists	8.60	1.298	9.00	
	General dentists	7.27	1.438	7.00	
	Non-professionals	7.00	2.000	7.00	
F	Periodontists	7.53	1.959	8.00	0.468*
	Restorative dentists	6.47	2.100	6.00	
	General dentists	6.73	1.870	7.00	
	Non-professionals	7.13	2.295	8.00	
G	Periodontists	5.00	2.236	6.00	0.002*
	Restorative dentists	4.60	2.354	6.00	
	General dentists	5.20	2.042	5.00	
	Non-professionals	7.80	2.210	8.00	
H	Periodontists	7.33	1.291	7.00	0.605*
	Restorative dentists	7.00	1.558	7.00	
	General dentists	7.00	1.964	7.00	
	Non-professionals	7.67	2.193	8.00	
I	Periodontists	7.00	1.604	7.00	0.005*
	Restorative dentists	6.13	1.767	6.00	
	General dentists	6.33	1.397	7.00	
	Non-professionals	8.00	2.035	8.00	
J	Periodontists	4.87	2.356	4.00	0.018*
	Restorative dentists	4.13	2.066	4.00	
	General dentists	4.60	1.682	5.00	
	Non-professionals	6.73	2.154	6.00	

*Kruskal-Wallis test

The difference in opinions of the rater groups was not significant for photographs B (*p*=0.176), C (*p*= 0.322), D (*p*= 2.332), F (*p*=0.468), and H (*p*= 0.605).

Within-group comparison of the scores given by the rater groups to different photographs (Table 6) showed a significant difference in the group of periodontists (*p*< 0.001), restorative dentists (*p*< 0.001), and also general dentists (*p*< 0.001); such that all of them gave the highest score to photograph A and the lowest score to photograph J. The difference in this regard was not significant in the group of non-professionals (*p*= 0.260).

**Table 6:** Within-group comparison of scores given by the four rater groups to different photographs

Rater group	Photograph	Mean	Maximum	Minimum	*p* Value
Periodontists	A	9.06	10.00	8.00	< 0.001
	B	8.20	10.00	6.00	
	C	8.20	10.00	5.00	
	D	7.60	10.00	5.00	
	E	8.66	10.00	6.00	
	F	7.53	10.00	5.00	
	G	5.00	8.00	1.00	
	H	7.33	9.00	5.00	
	I	7.00	10.00	5.00	
	J	4.86	8.00	1.00	
	Total	7.34	10.00	1.00	
Restorative dentists	A	9.33	10.00	7.00	< 0.001
	B	8.66	10.00	6.00	
	C	8.33	10.00	5.00	
	D	7.20	10.00	4.00	
	E	8.60	10.00	6.00	
	F	6.46	10.00	3.00	
	G	4.60	9.00	1.00	
	H	7.00	9.00	3.00	
	I	6.13	9.00	3.00	
	J	4.13	10.00	1.00	
	Total	7.04	10.00	1.00	
General dentists	A	7.73	10.00	5.00	< 0.001
	B	7.13	10.00	2.00	
	C	7.06	10.00	2.00	
	D	6.26	9.00	1.00	
	E	7.26	9.00	4.00	
	F	6.73	9.00	3.00	
	G	5.20	8.00	1.00	
	H	7.00	10.00	2.00	
	I	6.33	8.00	2.00	
	J	4.60	8.00	2.00	
	Total	6.53	10.00	1.00	
Non-professionals	A	8.73	10.00	7.00	0.260
	B	7.53	10.00	4.00	
	C	7.60	10.00	5.00	
	D	7.06	10.00	2.00	
	E	7.00	10.00	3.00	
	F	7.13	8.00	2.00	
	G	7.80	9.00	3.00	
	H	7.66	10.00	3.00	
	I	8.00	10.00	2.00	
	J	6.73	10.00	4.00	
	Total	7.52	10.00	2.00	

Since there was a significant difference in the opinions of the four rater groups for photographs A, E, G, I, and J (*p*< 0.05), pairwise comparisons were carried out between each pair of raters for each of the aforementioned photographs, which showed significant differences between general dentists and periodontists (*p*= 0.027), and general dentists and restorative dentists (*p*= 0.005) for photograph A, non-professionals and restorative dentists (*p*= 0.011), non-professionals and periodontists (*p*= 0.008), general dentists and restorative dentists (*p*= 0.015), and general dentists and periodontists (*p*= 0.012) for photograph E, restorative dentists and non-professionals (*p*= 0.001), periodontists and non-professionals (*p*= 0.003), and general dentists and non-professionals (*p*= 0.004) for photograph G, restorative dentists and non-professionals (*p*= 0.001) and general dentists and non-professionals (*p*=0.004) for photograph I, and restorative dentists and non-professionals (*p*= 0.003), general dentists and non-professionals (*p*=0.016), and periodontists and non-professionals (*p*= 0.032) for photograph J.

To check the intra-examiner reliability, all the images were printed with the same level of saturation and similar dimensions, color, thickness, and paper type. The photographs were given to 10 raters, who were randomly selected, after two weeks to rate the images again under natural daylight. The interclass correlation coefficient for each picture was calculated which revealed that the intra-examiner reliability was acceptable. 

## Discussion

This study assessed the effects of the shape and position of the gingival margin of maxillary anterior teeth on the perception of smile aesthetics by periodontists, restorative dentists, general dentists, and non-professionals.

The total number of female raters was significantly higher than male raters, and females generally gave a higher score to all photographs; however, this difference was only significant for photographs A, E, and J. Males were more sensitive to changes in the shape and position of the gingival zenith than females. In contrast, Alhammadi *et al*. [ 17 ] reported that males detected changes in occlusogingival height significantly better than females. Alhammadi *et al*. [ 17 ] investigated some other effective features in addition to gingival zenith but in the case of gingival zenith; they only paid attention to its occlusogingival position. Perhaps these differences in the design of the two studies, explain the contradictory results.

In the present study, the frequency of raters over 30 years of age was significantly higher than the number of raters younger than 30 years. A significant difference also existed in the mean age of the four rater groups, and the highest mean age belonged to periodontists while the lowest mean age belonged to general dentists. Older raters gave a higher score to photographs A to F, while younger raters gave a higher score to photographs G to J. However, only the difference for photograph J was significant. Since photographs G, H, I, and J indicated asymmetry in the position and shape of the gingival zenith of the central incisor only, the lateral incisor only, the canine only, and all three anterior teeth, it may be concluded that older raters were more sensitive to the detection of asymmetry; however, it should be noted that this difference was only significant for asymmetry in all three teeth (photograph J). 

The present results revealed a significant difference in the opinions of the four rater groups in photographs A, E, G, I, and J. Pairwise comparisons revealed that restorative dentists gave the highest score to photograph A and the lowest score to photographs J and I, compared with other raters. General dentists gave the lowest score to photograph A, and non-professionals gave the highest score to photographs J and I, compared with other rater groups. In all four rater groups, the highest scores were given to photograph A, and the lowest to photograph J. The difference in this regard was significant in the group of periodontists, restorative dentists, and general dentists, but not in non-professionals. The scores given to photograph B were lower than the scores given to photograph A by the raters, but this difference was not significant in any rater group. This result was not in line with the findings of Machado *et al*. [ 18 ]. In the present study, the change in gingival zenith of the lateral incisor in photograph B was 1mm, while this value was 0.4mm in the study by Machado *et al*. [ 18 ]. The present results were in agreement with the findings of Kokich *et al*. [ 19 ] since in their study non-professionals did not notice the occlusogingival changes in the position of the gingival zenith of the lateral incisor by 1 to 3mm. In the present study, the magnitude of all changes was 1mm. Kokich *et al*. [ 19 ] evaluated the change in position of gingival margin of the maxillary central incisor relative to the opposing tooth and noticed that orthodontists were more sensitive in the detection of 0.mm discrepancies, while non-professionals were less sensitive and only noticed changes greater than 2mm. 

In the present study, the scores given to symmetrical changes were higher than those given to asymmetrical changes, which was in accordance with the results of Nomura *et al*. [ 16 ]. They only evaluated changes in the occlusogingival height of the gingival zenith. Also, Alomari *et al*. [ 20 ] indicated that mesial shift of the gingival zenith unilaterally was rated as unattractive by both dentists and patients. In their study, different parameters related to smile aesthetics were evaluated in addition to the gingival zenith. 

It is believed that asymmetry is less attractive than symmetrical changes and is more easily detected. Kokich *et al*. [ 19 ] compared the perception scores of dentists and non-professionals using dental photographs with symmetrical and asymmetrical changes and reported that when the gingival margin was altered symmetrically, none of the three rater groups of orthodontists, general dentists, or non-professionals could detect the differences among different levels of gingival margin disharmony. However, it should be noted that the magnitude of asymmetry is also important, and Alhammadi *et al*. [ 17 ] stated that general dentists did not notice asymmetry by up to 0.5 mm. 

Comparison of the results obtained from photographs G and H revealed that periodontists, restorative dentists, and general dentists were significantly more sensitive to changes in photograph G than photograph H. Thus, it may be concluded that maxillary central incisors are more esthetically important to dentists than lateral incisors; this difference was not significant in the group of non-professionals. Machado *et al*. [ 18 ] reported that central incisors had greater effect than lateral incisors on the opinion of orthodontists and general dentists regarding smile aesthetics; the present findings were in line with their results. Machado *et al*. [ 18 ] evaluated images of maxillary central and lateral incisors altered by 0.5, 1, and 1.5 mm. The present results were also in agreement with the findings of Nomura *et al*. [ 16 ] who showed the effect of changes of 1 mm in the central and lateral incisors on the aesthetic score. 

Photograph I in the present study was used to assess the significance of canines’ gingival margin in smile aesthetic, in comparison with central and lateral incisors. Comparison of the results obtained from photograph I with G and H revealed no significant difference in any rater groups, indicating that canines’ gingival margin had no significant effect on smile attractiveness compared with central and lateral incisors. 

Unlike the present results, Pinho *et al*. [ 21 ] found that asymmetry by 2 mm in a canine tooth had no significant effect on perception of smile aesthetics. They explained that the negative effect of maxillary canine asymmetry on aesthetics is more negligible than lateral incisor asymmetry. In this comparison, the central incisors, the teeth closest to the midline, show the greatest need for symmetry. In the present study, the difference in scores given by the non-professionals to different photographs was not significant; thus, their aesthetic preferences could not be identified. 

The differences in the opinion of dentists and non-professionals, as shown in the present study, highlight the significance of focusing on patient expectations and explaining to them the differences between ideal and clinically acceptable, and the fact that the ideal state may not be achieved in all cases. Also, the significant effect of cultural issues on aesthetic preferences highlights the need for further investigations in this respect in different populations [ 22 ]. 

One of the current study's strengths was its evaluation of the gingival margin's impact on smile aesthetics, an issue that has not received sufficient attention in the literature. The other strength was that the raters were prohibited from placing the photographs side by side for comparison. 

The primary limitations of this study were the small sample size and the inability to include an equal number of males and females in each group. Future research with larger sample size is needed to assess the impact of various parameters and their changes by 0.5, 1.5, and 2 mm on the aesthetic judgments of different rater groups, providing a clearer understanding of this subject. Additionally, to explore potential differences between males and females in each group, future studies should aim to maintain a balanced gender distribution. It should be noted that numerous parameters can influence the aesthetics of a smile. This study, however, only assessed the effects of the shape and location of the gingival margin of maxillary anterior teeth. Future studies can evaluate the combination of different parameters that may impact smile aesthetics. 

## Conclusion

Periodontists, restorative dentists, general dentists and non-professionals had noticeably different opinions on how the shape and position of the gingival margin of maxillary anterior teeth affect the perception of smile aesthetics. This highlights the importance of achieving an interdisciplinary agreement before performing procedures like gingivectomy or aesthetic crown lengthening to ensure consistent and optimal results. It is essential to thoroughly discuss surgical procedures with patients prior to making any decisions, as non-experts often have different perceptions of smile aesthetics compared to the dental professionals. Before doing any operations on the gingiva, patients have to know how the shape of gingival margins is going to be changed after surgery to avoid any improper expectations.
